# miR-281, an abundant midgut-specific miRNA of the vector mosquito *Aedes albopictus* enhances dengue virus replication

**DOI:** 10.1186/s13071-014-0488-4

**Published:** 2014-10-22

**Authors:** Yanhe Zhou, Yanxia Liu, Hui Yan, Yiji Li, Hao Zhang, Jiabao Xu, Santhosh Puthiyakunnon, Xiaoguang Chen

**Affiliations:** Key Laboratory of Prevention and Control for Emerging Infectious Diseases of Guangdong, Department of Pathogen Biology, School of Public Health and Tropical Medicine, Southern Medical University, Guangzhou, China

**Keywords:** *Aedes albopictus*, miRNA, Dengue virus, Pathogen-host interaction

## Abstract

**Background:**

Emerging evidence indicates that microRNAs (miRNAs) are involved in host-virus interaction. We previously reported that some miRNAs were differentially expressed in sugar-fed and blood-fed females of *Aedes albopictus* (*Ae. albopictus*). Here, we analysis the role in the host-virus system of an abundant midgut-specific miRNA in the mosquito *Ae. albopictus*.

**Methods:**

The expression profiles of miR-281 in different body parts of *Ae. albopictus* and following dengue virus infection were determined using RT-qPCR and Northern blot. miR-281 mimics, antagomiRs and corresponding negative controls were designed and their overexpression and knock-down efficiency were analyzed by qRT-PCR after transfecting the mosquito cell lines C6/36, and also by injecting female mosquitoes. Dengue virus serotype-2 (DENV-2) viral genomic RNA abundance was determined by RT-qPCR. The levels of DENV-2 E protein were detected using Western blot. Virus titers were tested using TCID_50_. RNAhybrid was used to predict targets of miR-281 in the DENV-2 genome. The EGFP plasmid-based reporter system was used to investigate the interaction between miR-281 and the predicted binding site in the C6/36 cell line.

**Results:**

miR-281 is specifically expressed in the female midgut where dengue virus first invades. After DENV-2 infection, this miRNA is up-regulated in response to viral infection. Functional intervention analyses in vitro with specifically designed miR-281 mimics and corresponding antagomiRs indicated that miR-281 enhances DENV-2 viral replication. Further depletion of miR-281 in female mosquitoes by injection of its specific antagomiRs led to a significant reduction in DENV-2 abundance. The interaction between miR-281 and its predicted target sequence, the DENV-2 genomic 5*'*-untranslated region (UTR), is confirmed in the context of a plasmid-based reporter system.

**Conclusion:**

These findings confirm that miR-281, an abundant midgut-specific miRNA, facilitates DENV-2 replication.

**Electronic supplementary material:**

The online version of this article (doi:10.1186/s13071-014-0488-4) contains supplementary material, which is available to authorized users.

## Background

Dengue is currently one of the most serious world-wide public health problems [1]. An estimated 50 million dengue infections occur annually, and approximately 2.5 billion people live in dengue-endemic countries [2]. With global warming, uncontrolled urbanization and international travel, the economic burden of dengue-related illnesses increases every year [3,4]. Dengue virus (DENV) belongs to the genus *Flavivirus* of the family *Flaviviridae*. It is an enveloped, single-stranded, positive-sense RNA virus (+ssRNA) with a genome size of approximately 11,000 nucleotides. The genome of DENV contains a single open reading frame (ORF) flanked by conserved 5′ and 3′untranslated regions (UTRs) [5]. Transmitted primarily by *Aedes aegypti* (*Ae. aegypti*) and *Aedes albopictus* (*Ae. albopictus*), DENV is the most prevalent mosquito-borne virus [6]. Although the global burden of DENV represents a growing challenge to public health, no specific therapeutics or licensed vaccines against DENV are yet available. There is currently no better alternative than vector control to reduce or prevent dengue virus transmission [7]. Thus, understanding the viral and host determinants governing DENV replication can provide novel targets for mosquito vector control strategies.

MicroRNAs (miRNAs) are an abundant group of endogenous non-protein-coding small RNAs of ~22 nucleotides that post-transcriptionally regulate gene expression. They have been reported to play an important role in the regulation of embryonic development, apoptosis, tumorigenesis and immunity [8]. Recently, miRNAs were suggested to be involved in host-virus interaction. On one hand, miRNAs act in an antiviral role. Several miRNAs, including miR-28, miR-125b, miR-150, miR-223, and miR-382, have been reported to inhibit HIV-1 replication via directly targeting the viral genome [9]. A cellular miRNA, miR-32, was found to effectively restrict the accumulation of the retrovirus primate foamy virus type 1 in human cells [10]. On the other hand, cellular miRNAs can also benefit viral replication. It has been suggested that MiR-10a* up-regulates coxsackie virus B3 biosynthesis by targeting the 3D-coding sequence [11]. MiR-122, a liver-specific miRNA, has been reported to target two adjacent sites within the 5′-UTR of the HCV genomes to facilitate viral replication [12].

Recently, mosquito miRNAs have also been reported to positively or negatively modulate the host response to pathogen infection. Two miRNAs of the mosquito *Culex quinquefasciatus* (*C.quinquefasciatus*) showed significant changes in expression levels following WNV infection, implying potential roles for mosquito miRNAs in viral infection [13]. The knock-down of two important proteins in miRNA biogenesis, Dicer1 and Argonaute1, led to increased sensitivity to *Plasmodium* infection in *Anopheles gambiae*, supporting an involvement of miRNAs in the defense reaction [14]. However, relatively little is known about the interactions between mosquito miRNAs and dengue virus.

We previously reported that some miRNAs were differentially expressed in sugar-fed and blood-fed females of *Ae. albopictus* [15]. In this paper, we investigate the role of an abundant and tissue-specific miRNA of *Ae. albopictus* in DENV viral replication.

## Methods

### *Ae. albopictus* rearing and cell culture maintenance

*Ae. albopictus* were provided with 10% glucose solution and reared at 28°C, 80% relative humidity, with a 16–8 h light–dark photoperiod as reported previously [16]. Then, 3- to 5-day-old adult mosquitoes were fed on the blood of healthy white rats to produce eggs. Because female pupae are bigger than males, female pupae could be selected by size after hatching, and they were transferred to plastic cups covered with nets and allowed to emerge. The *Ae. albopictus* cell line C6/36 was grown at 28°C in Dulbecco’s modified Eagle’s medium (DMEM) (Invitrogen, Carlsbad, CA, USA) with 10% fetal calf serum (Gibco, New York, NY), 2 mM L-glutamine, 50 U/ml penicillin and 50 μg/L streptomycin (Gibco).

### Tissue dissection and RNA isolation

Dissections were conducted on ice in cold PBS (Gibco) and kept on ice. For analysis of the expression profiling of miR-281 across body parts, dissections were performed on female adults between 3 and 5 days old. Mosquitoes were separated into parts: head, thorax, midgut and the remaining part as previously described. The remaining part, which includes the ovaries, the fat body, muscles and abdominal cuticle, was labeled as “leftover” [14]. Total RNA isolation was carried out using a mirVana miRNA isolation kit (Ambion by Life Technologies, TX, USA) throughout this study.

### DENV-2 virus propagation and infection

The New Guinea C strain (NGC) of DENV-2 was propagated in C6/36 cells. The C6/36 cell cultures grown on 25-cm^2^ flasks at 90% confluence were infected at a multiplicity of infection (MOI) of 5 and incubated at 37°C with 5% CO_2_ for 4 days. Virus titer was determined by tissue-culture infectious dose 50 (TCID_50_) to be 1 × 10^7^/ml on C6/36 cells. For oral infection of mosquitoes throughout the study, 3- to 5-day-old female mosquitoes were deprived of sugar for 24 hrs prior to infection. To prepare 1 ml infectious blood meals, 500 μl virus stock was mixed 1:1 with 500 μl commercial human blood. The media of uninfected C6/36 cells were cultured under similar conditions and served as mock-infected controls. The blood meals were maintained for 30 min in a 37°C water bath prior to feeding mosquitoes [17]. For C6/36 cell infections in the study, cells were infected with DENV-2 at MOI = 0.01 and incubated at 37°C with 5% CO_2_ for 1 hr. The supernatant was then replaced by DMEM containing 2% fetal calf serum, and the cells were cultured at 28°C.

### Northern blot hybridizations

Northern blots were carried out as described by Mead and Tu [18]. Briefly, total RNA were loaded onto 15% denaturing poly-acrylamide gels and run beside 19- and 23-nucleotide-long ssDNA markers. The RNA gels were transferred to Bright-Star-Plus nylon membranes (Ambion), crosslinked using a UV crosslinker, prehybridized, and then hybridized overnight in the ULTRAhyb-Oligo Hybridization Buffer (Ambion) with the appropriate DIG-labeled probe at 42°C. Wash conditions were the same as described in Mead et al. [18]. Antisense 5′digoxigenin-labeled miRCURYLNA probes were purchased from Exiqon (Vedbaek, Den-mark). The loading control was used as previously described [15,19].

### Functional interventional studies of miR-281

miRNA mimics are chemically modified double-stranded RNAs that mimic mature endogenous miRNA while antagomiRs are chemically modified, cholesterol-conjugated single-stranded RNA analogues complementary to miRNAs. AntagomiRs differ from normal antisense oligonucleotide (ASO) by complete 2′-O-methylation of sugar, phosphorothioate backbone and a cholesterol-moiety at 3′end [20]. The silencing of endogenous miRNAs by antagomiRs is specific, efficient and longlasting. To overexpress miR-281, a miR-281 mimic (sense strand 5′-AAGAGAGCUAUCCGUCGACAGU-3′; anti-strand 5′-UGUCGACGGAUAGCUCUCUUUU-3′) and a negative control mimic (an unrelated mimic, sense strand 5′-UUCUCCGAACGUGUCACGUTT-3′; anti-strand 5′-ACGUGACACGUUCGGAGAATT-3′) were used [21,22]. To deplete the expression of miR-281, anantagomiR consisting of the reverse complement of miR-281, termed antagomiR-281 (5′-ACUGUCGACGGAUAGCUCUCUU-3′), and a scrambled control antagomiR (5′-UUGUACUACACAAAAGUACUG-3′) were used. MiRNA mimics and antagomiRs used in this study were synthesized by GenePharma (Shanghai, China). For cell transfection, C6/36 cells were seeded at a density of 5.5 × 10^6^ /well in a 6-well plate 12 hrs before transfection. Cells were transfected with a transfection mixture containing 100 pmol miRNAs (mimic, antagomiR, control) and 8 μl Cellfectin II (Invitrogen) in 2 ml serum-free DMEM per well. Normal cells without any treatment served as mock controls. Cells were collected 24 hrs after transfection to validate the expression of miR-281 after overexpression or knock-down. For functional analysis of the role of miR-281 in regulating DENV-2, the transfected cells were infected with DENV-2 (MOI of 0.01) at 24 hrs post-transfection and harvested at 48 hours post-infection (hpi). To knock down miR-281 in female adults, 3- to 5-day-old female mosquitoes were cold-anesthetized and injected into the thorax with miR-281 antagomiRs, each with 0.5 μl at a final concentration of 300 μM. After injection, mosquitoes were then transferred to small plastic cups with mosquito nets and allowed to recover. Female mosquitoes injected with the same volume of control antagomiR served as negative controls while normal adults without injection served as mock-injected controls. Both injected and mock-injected mosquitoes were maintained under the same conditions. To test the knock-down efficiency of miR-281, total RNA was extracted from individual mosquitoes and midguts at 48 hrs post-injection. To investigate the knock down effect of miR-281 on DENV replication, the injected mosquitoes were fed an infectious blood meal as described above at 48 hrs post-injection. Total RNA was extracted from individual mosquitoes at 1, 4 and 7 days post-infection (dpi) to detect DENV-2 viral abundance.

### RT-qPCR

The miScript PCR system was used to quantify gene expression in this study. The system includes the miScript Reverse Transcription Kit (Qiagen, Duesseldorf, Germany) and the miScript SYBR Green PCR kit (Qiagen). Expression levels of miR-281 were assayed following the miScript SYBR System Handbook. For miR-281 expression detection, PolyA tails were added to miRNAs using the polyA polymerase provided by the kit. The polyadenylated miRNAs were converted to cDNA by reverse transcriptase using random primers and oligo-dT. Reverse transcription was performed at 37°C for 1 h and then 95°C for 5 min. Real-time PCR was performed using the cDNAs as template and the SYBR Green for detection. The forward primer for miR-281 was synthesized by Invitrogen, and a universal 3′-end primer was provided by the miScript kit. The PCR conditions were as follows: 95°C for 15 min and then 35 cycles at 94°C for 15 s, 55°C for 30 s, and 72°C for 30 s. 5S rRNA was used as the internal control. For the levels of EGFP and DENV-2 genomic (gRNA) expression detection, cDNAs were synthesized, and previously described specific primers for the EGFP gene and DENV-2 gRNA (NS1 region) were used for qPCR as described above [23]. The expression levels of the *Ae. albopictus* rpS7 (ribosomal protein 7) gene were used as an internal control for DENV-2 gRNA. Primers for rpS7 were synthesized as previously reported [16]. Blasticidin resistance gene was used as an internal control for EGFP detection. RT-qPCR was performed on a MX3005P TM Real Time PCR System (Stratagene, CA, USA). All the qPCR primers used in this study are shown in Additional file 1: Table S1.

### Western blot analysis

Total proteins were extracted with the Radio Immunoprecipitation Assay (RIPA) Lysis Buffer (Sangon, Shanghai, China) following the manufacturer’s instructions. The protein concentration was determined using the BCA Protein Assay Kit (Sangon). Total proteins were separated by 10% sodium dodecyl sulfate-polyacrylamide gelelectrophoresis (SDS)-PAGE and then transferred to polyvinylidenedifluoride (PVDF) membranes. Membranes were blocked for 1 hr at room temperature with Tris–HCl buffer plus 0.5% Tween 20 (TBST) and 5% non-fat milk. Afterwards, the membranes were incubated overnight at 4°C with a mouse monoclonal antibody against the DENV-2 E protein, diluted 1/200 (Santa Cruz Biotechnology, CA, USA sc-65659). Membranes were then incubated with a 1:2,000 dilution of peroxidase-conjugated goat anti-mouse IgG (Proteintech Group, Inc., Chicago, IL) for 1 hr. Blots were visualized by an enhanced chemiluminescence reagent (Forevergen, Guangzhou, China). *Ae. albopictus* beta-actin was used as an internal control and detected as described above with a mouse pan-actin antibody, diluted 1:10000 (Santa Cruz, sc-2005).

### Titration of DENV-2 in supernatant of C6/36 cells

The culture supernatant of normal C6/36 cells and cells transfected with antagomiR-281 or control antagomiR at 48 hrs post-transfection were collected. Virus titers were calculated by log_10_TCID_50_ according to a previous report [24].

### MiRNA target studies

RNAhybrid was used to predicted targets of miR-281 in the DENV-2 genome according to the complementary sequence and minimum free energy (mfe) < −20.0 kcal/mole. To investigate the miR-281-target interaction, an EGFP fragment of 720 bp was cloned into EcoRV and XbaI sites in the pIB-V5-His expression vector (Invitrogen), resulting in a plasmid-based reporter termed pIB/EGFP. Based on pIB/EGFP, a 89-bp fragment (nt8-nt96) of the DENV-2 5′-UTR (GenBank accession number: M29095.1) and a mutant 5′-UTR that contains mutations in the predicted binding site of the miR-281 seed region were cloned upstream of the EGFP gene construct using the EcoRV restriction site in a plus-strand orientation, resulting in the constructs pIB/5UTR-EGFP and pIB/mut-5UTR-EGFP, respectively. For the transfection experiment, cells were seeded into each well in 24-well plate 12 hrs prior to transfection. pIB/5UTR-EGFP and pIB/mut-5UTR-EGFP were co-transfected with the miR-281 mimic or the control mimic [22]. Transcript levels of EGFP were analyzed by RT-qPCR 48 hrs post-transfection using blasticidin resistance gene as an internal control as described above.

### Statistical analysis

SPSS version 13.0 (SPSS Inc., Chicago, IL) was used for data analysis. Continuous variables with normal distribution are presented as the mean ± standard deviation (Mean ± SD). *T*-tests were used for comparison of the relative expression of miR-281 in response to DENV-2 infection. Analysis of variance (ANOVA) was used for comparison of virus titer, E protein intensity and RT-qPCR data except for the relative expression of miR-281 in response to DENV-2 infection. Asterisks indicate the statistical significance: *P <0.05, **P <0.01, ***P <0.001. Error bars show the standard deviation. All RT-qPCR results are representative of at least three independent experiments, each with three technical replicates.

### Ethical standards

All animal studies have been approved by The Institute Research Medical Ethics Committee of Southern Medical University. All human studies have been performed in accordance with the ethical standards laid down in the 1964 Declaration of Helsinki and its later amendments.

## Results

### MiR-281 is specifically expressed in the *Ae. albopictus* midgut and is regulated by DENV-2 infection

**Figure 1:**
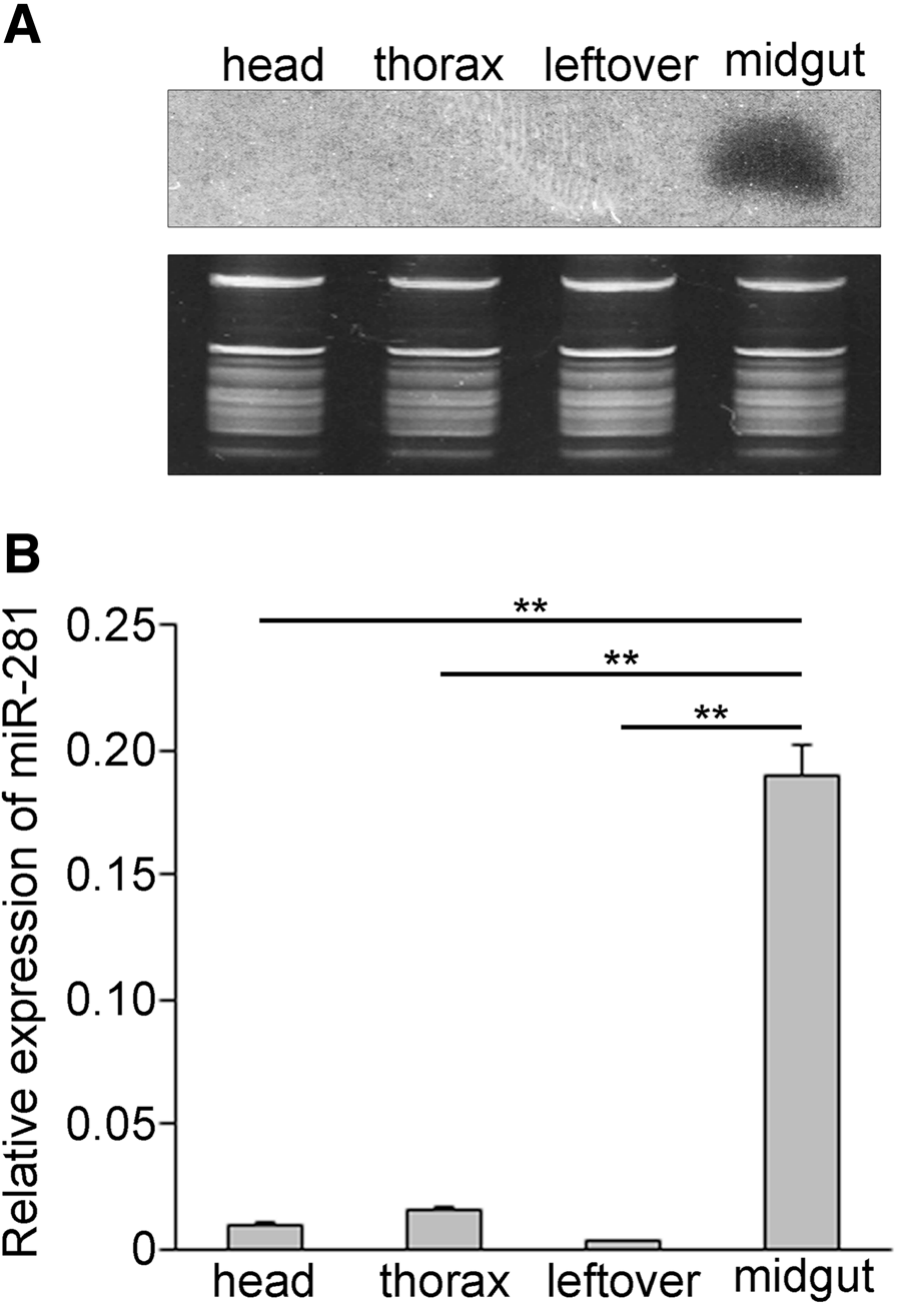
**Expression profiling of** ***Ae. albopictus*** **miR-281 across body parts. (A)** Northern blot hybridization of miR-281 expressed in body parts of 3- to 5-day-old female mosquitoes, showing the mature miRNA band (upper) using a probe specific to mature miR-281. Ethidium bromide staining of rRNA and tRNA is shown as a loading control (lower). **(B)** RT-qPCR analysis of miR-281 expressed in body parts of 3- to 5-day-old female mosquitoes. The relative expression of miR-281 was calculated as normalized to 5S rRNA. The RT-qPCR data were analyzed using the 2^-Δct^ method. Data shown represent three independent experiments with three triplicates each. *P* values were calculated using ANOVA.

**Figure 2:**
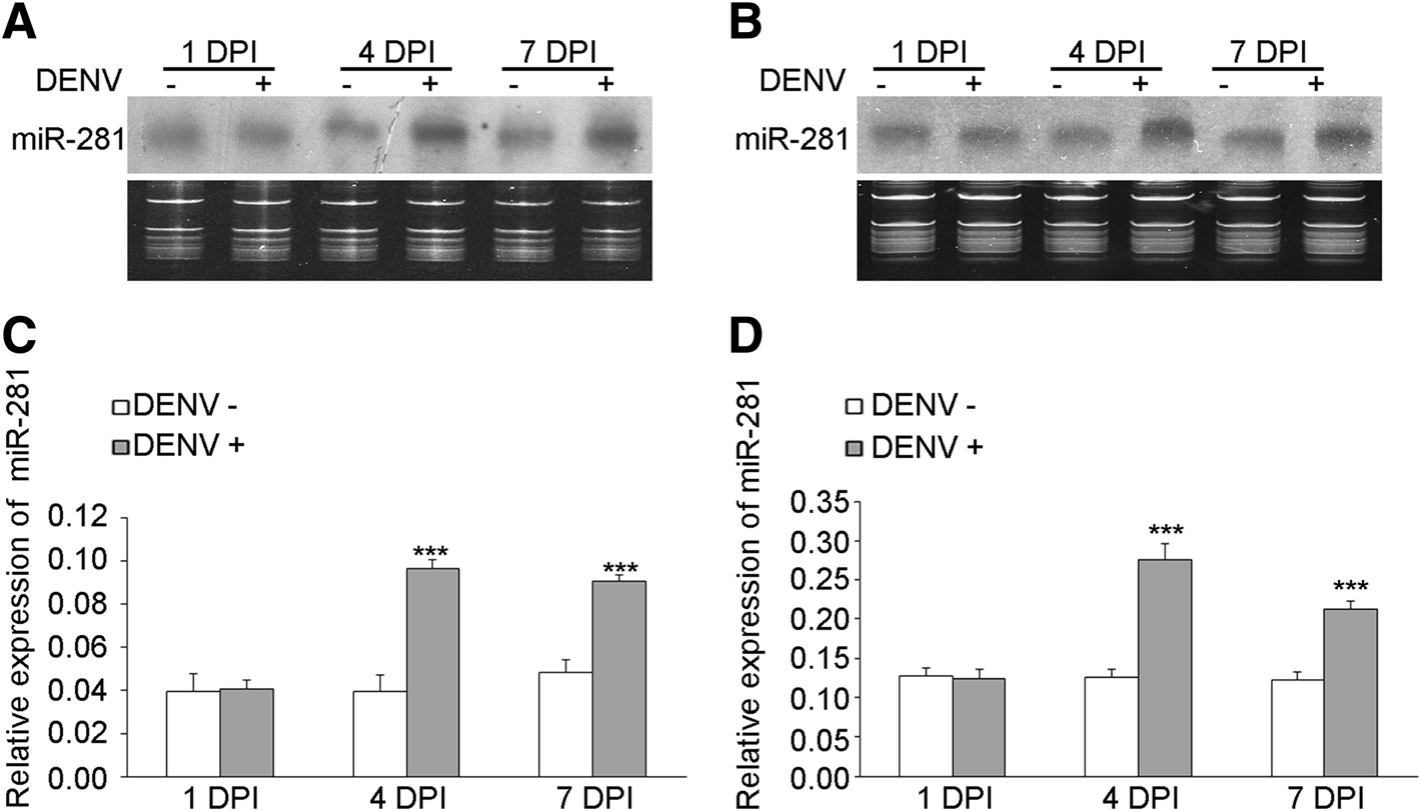
**Expression patterns of miR-281 in DENV-2-infected (DENV+) and uninfected (DENV−) female mosquitoes or midguts at 1, 4, and 7 dpi.** Northern blot analysis showing the expression patterns of miR-281 in response to DENV-2 at 1, 4 and 7 dpi in both mosquitoes **(A)** and midguts **(B)**. RT-qPCR analysis of the relative expression of miR-281 in response to DENV-2 at 1, 4 and 7 dpi in mosquitoes **(C)** and midguts **(D)**. The relative expression of miR-281 was calculated normalized to 5S rRNA. The RT-qPCR data were analyzed using the 2^-Δct^ method. Data shown represent three independent experiments with three triplicates each. *P* values were calculated using *t* tests.

**Figure 3:**
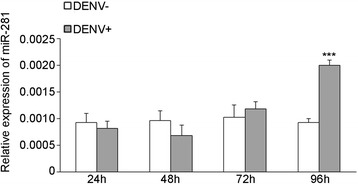
**RT-qPCR analyses of miR-281 expression at different time intervals following DENV-2 infection in C6/36 cells.** The relative expression of miR-281 was calculated normalized to 5S rRNA. The RT-qPCR data were analyzed using the 2^-Δct^ method. Data shown represent three independent experiments with three triplicates each. *P* values were calculated using *t* test.

We decided to focus on miR-281 because of its high expression in both sugar-fed and blood-fed female mosquitoes according to our previous study [15]. The expression profiles of miR-281 in body parts of *Ae. albopictus* were confirmed using total RNA extracted from different body parts of female mosquitos: the head, thorax, midgut and leftover. Based on the Northern blot analysis, we found that miR-281 was predominantly expressed in the midgut (Figure 1A). This result was confirmed by RT-qPCR analysis, which showed significantly higher levels (*F* =646.474, *P* <0.001) in the midgut compared to other body parts (Figure 1B). To explore whether the expression of miR-281 is part of the mosquito’s response to DENV-2 viral replication, expression profiles of miR-281 following dengue virus infection were determined by Northern blot at days 1, 4 and 7 post-infection. These time points were chosen according to the kinetics of DENV-2 midgut infection in the closely related spices *Ae. aegypti* and correspond to the very early stage of infection, the spread of the infection to neighboring cells and the infection of the entire organ, respectively [25]. Changes in miR-281 expression were not observed in either DENV-2-infected (DENV+) mosquitoes or DENV + midguts compared with levels in uninfected (DENV-) mosquitoes or DENV- midguts at 1 dpi. However, miR-281 expression increased in DENV + mosquitoes as well as in DENV + midguts when compared with DENV- mosquitoes and DENV- midguts at 4 dpi and 7 dpi. RT-qPCR analysis confirmed significant increases in miR-281 expression in both DENV-2 infected mosquitoes and midguts at 4 dpi and 7 dpi (all *P* <0.001), while no significant differences were seen at 1 dpi (all *P* >0.05) (Figure 2). Temporal expression profiles of miR-281 were also detected in the mosquito cell line C6/36 after DENV-2 infection and were seen to increase significantly at 96 hpi (*t* = −15.245, *P* <0.001) (Figure 3). These expression patterns suggested that miR-281 is specifically expressed in the mosquito midgut and up-regulated by DENV-2.

### MiR-281 positively regulates DENV viral replication in mosquito cells

**Figure 4:**
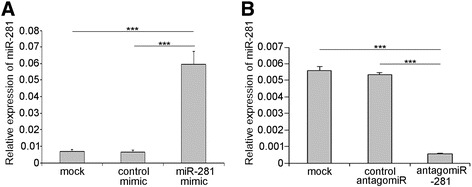
**Expression of miR-281 in C6/36 cells after transfection with mimics and antagomiRs. (A)** The relative expression of miR-281 increases significantly at 24 hrs post-transfection with the miR-281 mimic. **(B)** The relative miR-281 expression level is significantly reduced by transfection with the miR-281 antagomiRs at 24 hrs post-transfection. mock: normal cells; control mimic: cells transfected with control mimic; miR-281 mimic: cells transfected with miR-281 mimics; control antagomiR: cells transfected with control antagomiRs; antagomiR-281: cells transfected with miR-281 antagomiRs. The relative expression of miR-281 was calculated normalized to 5S rRNA. The RT-qPCR data were analyzed using the 2^-Δct^ method. Data shown represent three independent experiments with three triplicates each. *P* values were calculated using ANOVA.

**Figure 5:**
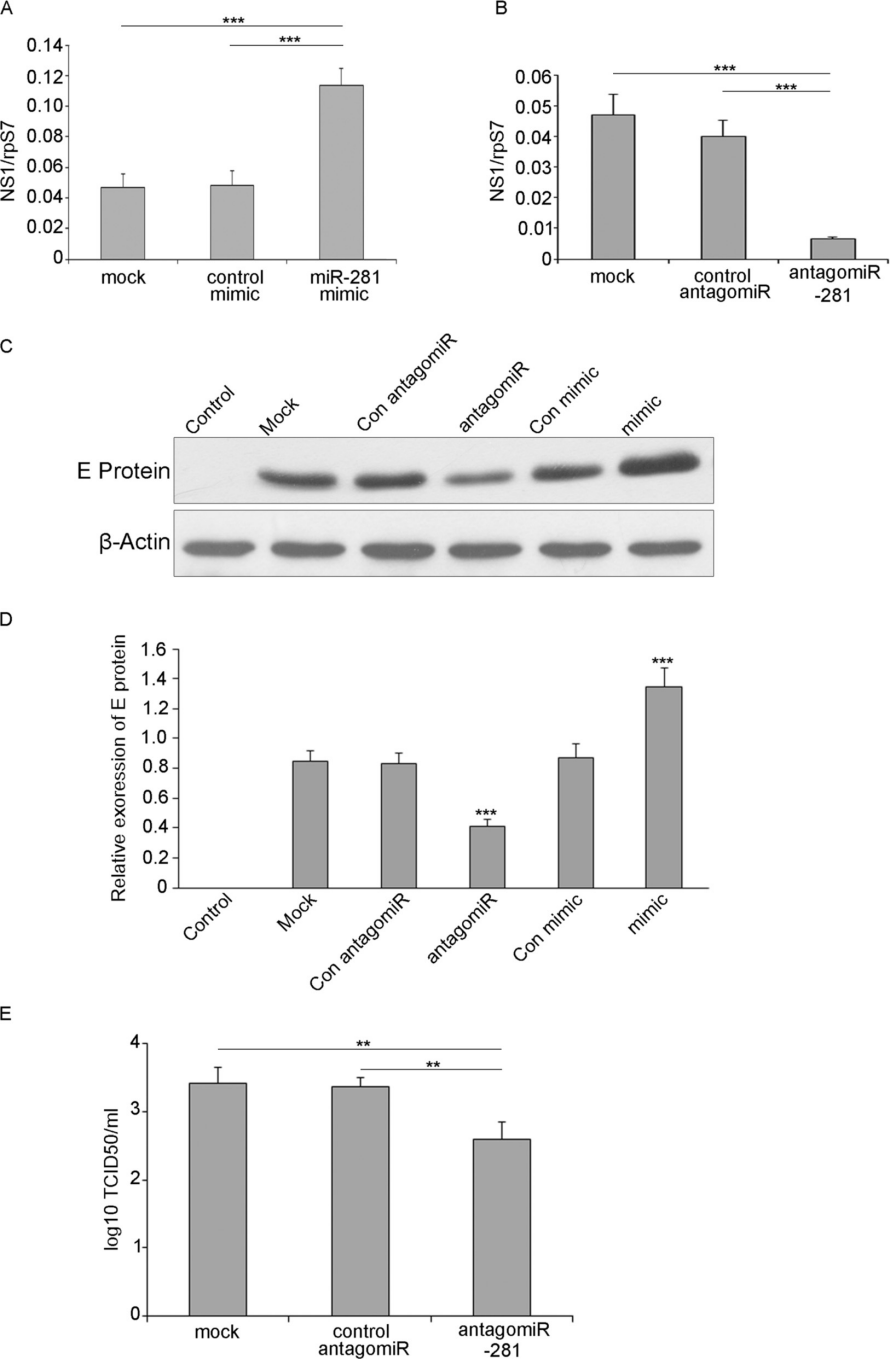
**MiR-281 enhances virus replication in C6/36 cells. (A)** miR-281 mimic treatment up-regulates DENV-2 gRNA. mock: normal cells + DENV-2; control mimic: control mimic + DENV-2; miR-281 mimic: miR-281 mimic + DENV-2. **(B)** antagomiR-281 treatment down-regulates DENV-2 gRNA. mock: normal cells + DENV-2; control antagomiR: control antagomiR + DENV-2; antagomiR-281: miR-281 antagomiR + DENV-2. The results of **(A) (B)** were analyzed using the 2^-Δct^ method. DENV-2 gRNA (NS1 region) was quantified by RT-qPCR and normalized to rpS7 gene. Data shown represent three independent experiments with three triplicates each. P values were calculated using ANOVA. **(C)** Western blot analyses of C6/36 cells treated as in **(A)** and **(B)** for the detection of DENV-2 E protein. The E protein level is up-regulated by the miR-281 mimic and down-regulated by the miR-281 antagomiR. Mosquito beta-actin was used as control for protein loading. Control: normal cells; Mock: normal cells + DENV-2; Con mimic: control mimic + DENV-2; mimic: miR-281 mimic + DENV-2; Con antagomiR: control antagomiR + DENV-2; antagomiR-281: miR-281 antagomiR + DENV-2. **(D)** Semiquantitative analysis of E protein levels. Data shown represent three independent experiments. The western blot band intensities were measured with ImageJ software. E protein levels were normalized to beta-actin levels. *P* values were calculated using ANOVA. **(E)** DENV-2 titers of cells treated as **(B)** were measured by TCID_50_ in C6/36 cells and were calculated by log_10_TCID_50_. Data shown represent three independent experiments with three triplicates each. *P* values were calculated using ANOVA.

**Figure 6:**
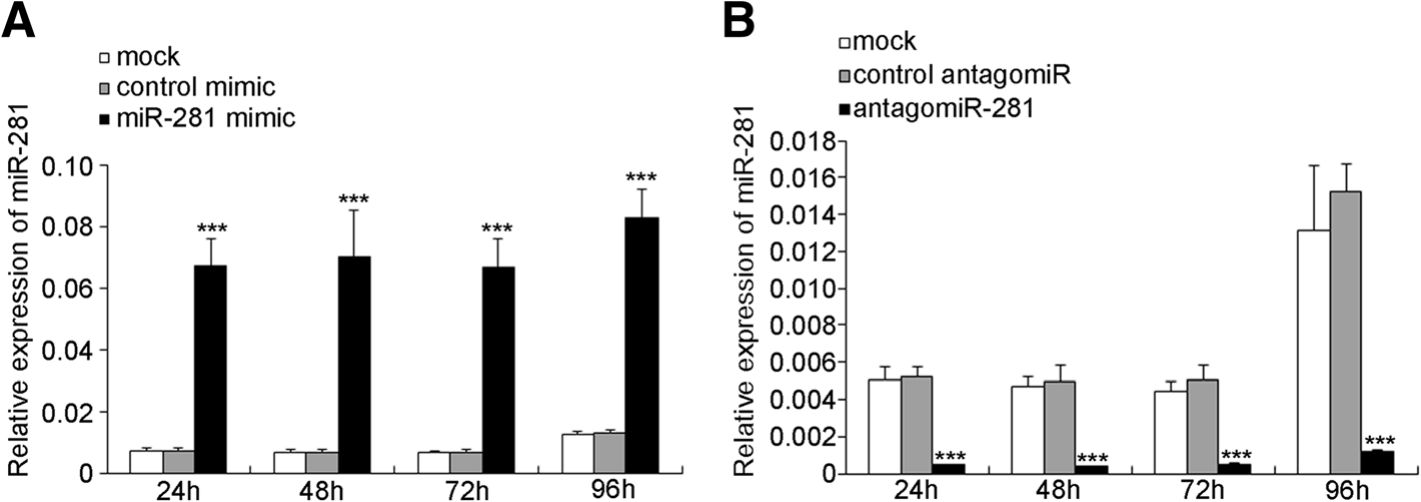
**RT-qPCR analyses of miR-281 expression at different time intervals after DENV-2 infection in cells previously transfected with miRNA mimics (A) or antagomiRs (B). (A)** mock: normal cells + DENV-2; control mimic: control mimic + DENV-2; miR-281 mimic: miR-281 mimic + DENV-2. **(B)** mock: normal cells + DENV-2; control antagomiR: control antagomiR + DENV-2; antagomiR-281: miR-281 antagomiR + DENV-2. The RT-qPCR data were analyzed using the 2^-Δct^ method. The relative expression of miR-281 was calculated normalized to 5S rRNA. Data shown represent three independent experiments with three triplicates each. *P* values were calculated using ANOVA

The expression patterns of miR-281 led us to investigate its effect on virus replication. The influence of miR-281 on DENV-2 viral replication was explored in the mosquito cell line C6/36. Cells were first transfected with mimics to achieve higher expression levels of miR-281 or antagomiRs to deplete the endogenous miR-281. The relative expression of miR-281 after overexpression and knock-down was evaluated by RT-qPCR at 24 hrs post-transfection. As expected, the expression of miR-281 was significantly higher in cells transfected with the miR-281 mimic compared with that in mock-transfected and control mimic-transfected cells (*F* =136.926, *P* <0.001) (Figure 4A). In contrast to the miR-281 mimic, miR-281 antagomiRs significantly down-regulated the expression of miR-281 (*F* =931.631, *P* <0.001) (Figure 4B). Subsequently, cells were infected with DENV-2, and the viral gRNA abundance was determined by RT-qPCR at 48 hpi. Compared with the levels in the mock-transfected and negative mimic-transfected controls, replication of DENV-2 gRNA was significantly increased (*F* =46.581, *P* <0.001) in the cells transfected with the miR-281 mimic (Figure 5A). In contrast, a significant reduction (*F* =54.594, *P* <0.001) of viral gRNA was detected in the antagomiR-281 transfected cells when compared with the mock-transfected and negative mimic-transfected controls (Figure 5B). Western blot analysis was also performed using a DENV-2 E protein-specific polyclonal antibody. DENV-2 E protein levels were significantly increased in cells overexpressing miR-281, whereas the E protein level in the miR-281-depleted cells was greatly reduced (*F* =110.218, *P* <0.001) (Figure 5C, D). The DENV-2 titers in the supernatant of C6/36 cells were further tested to investigate whether miR-281 affected DENV-2 infectivity. Compared with the other two control groups, DENV-2 titers were significantly decreased in the miR-281 depletion group (*F* =14.981, *P* <0.01) (Figure 5E). These results indicated that miR-281 positively regulates DENV replication in mosquito cells. More evidence was shown in Figure 6. DENV-2 infection did not affect or mimic antagomiR treatment in C6/36 cells.

### Knock-down of miR-281 down-regulates DENV viral abundance in vivo

**Figure 7:**
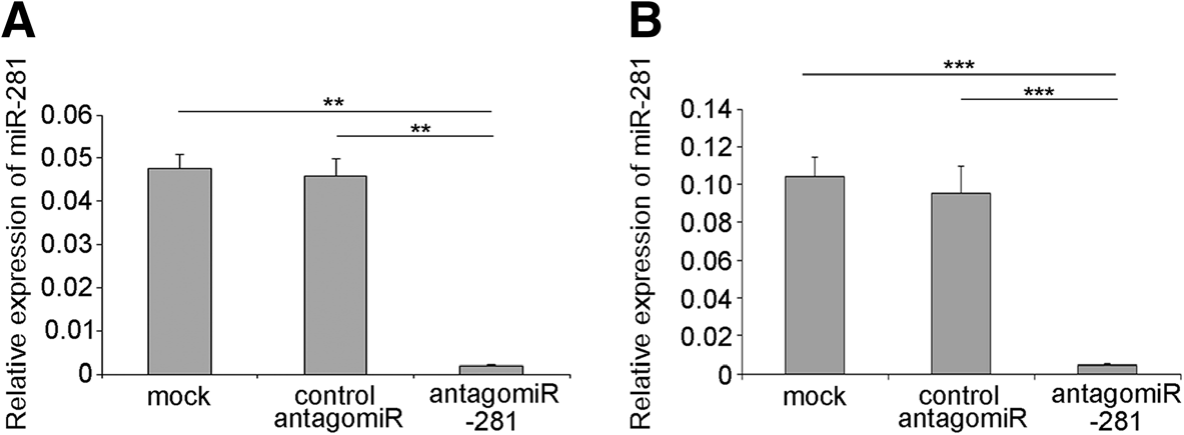
**Expression of miR-281 in female mosquitoes and midguts at 48 hrs after injection of miR-281 antagomiRs. (A)** RT-qPCR analysis of the relative expression of miR-281 in female mosquitoes, showing a significant decrease in antagomiR-281-injected mosquitoes compared to that of mock-injected (normal female mosquitoes without injection) and control antagomiR injected mosquitoes. **(B)** The relative expression of miR-281 in mosquitoes was significantly decreased in the midguts of antagomiR-281-injected mosquitoes. Midguts were dissected from mosquitoes treated as in **(A)**. The RT-qPCR data were analyzed using the 2^-Δct^ method. Data shown represent five (mosquito) or three (midgut) independent experiments with three triplicates. *P* values are from ANOVA.

**Figure 8:**
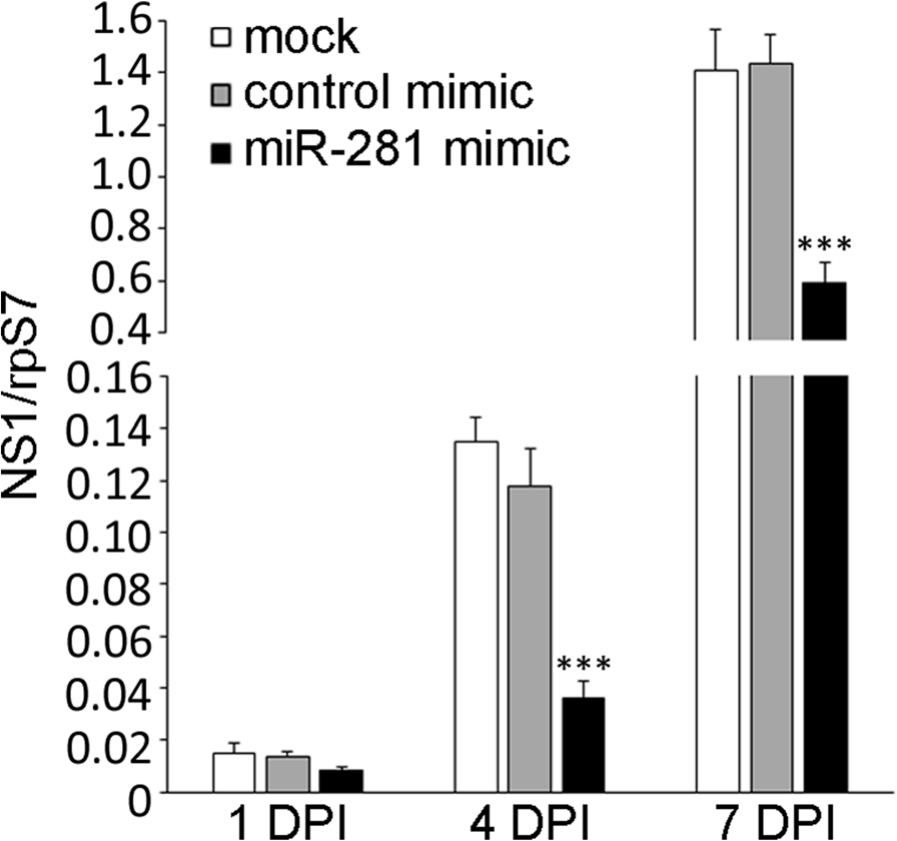
**Depletion of miR-281 drastically affects DENV-2 abundance in female mosquitoes.** Knock-down of miR-281 expression by miR-281 antagomiRs significantly down-regulates DENV-2 gRNA. DENV-2 gRNA was quantified by RT-qPCR and normalized to the rpS7 gene. The RT-qPCR data were analyzed using the 2^-Δct^ method. Data shown represent three independent experiments with three triplicates each. *P* values were calculated using ANOVA.

**Figure 9:**
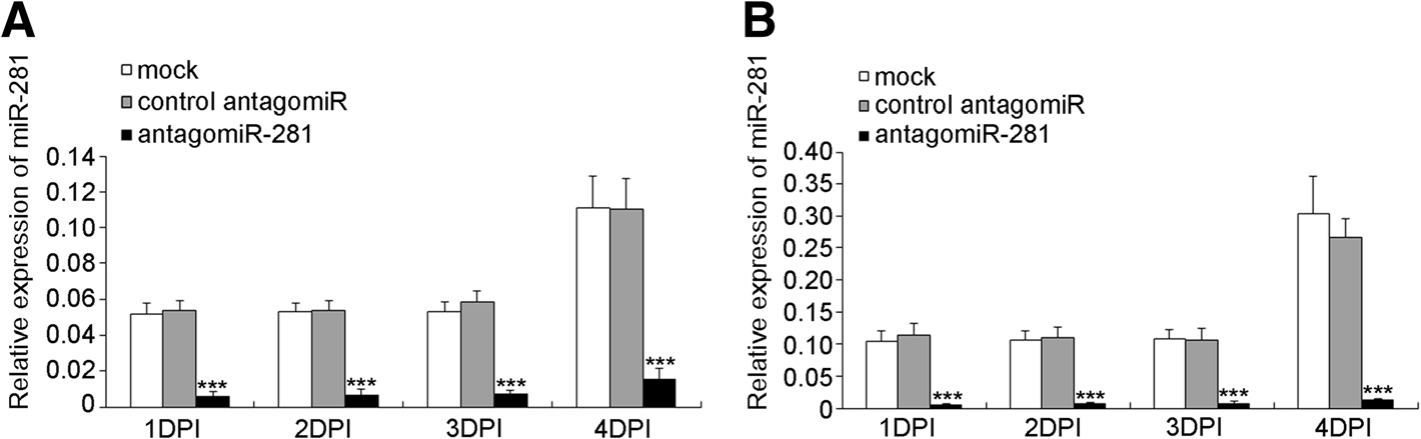
**RT-qPCR analyses of miR-281 expression at different time intervals following DENV-2 infection in female mosquitoes (A) or midguts (B) previously injected with antagomiRs.** mock: normal cells + DENV-2; control antagomiR: control antagomiR + DENV-2; antagomiR-281: miR-281 antagomiR + DENV-2. The relative expression of miR-281 was calculated normalized to 5S rRNA. The RT-qPCR data were analyzed using the 2^-Δct^ method. Data shown represent three independent experiments with three triplicates each. *P* values were calculated using ANOVA.

Considering the evidence that miR-281 positively regulates DENV-2 replication in C6/36 cells, we examined whether a similar effect could be reproduced in *Ae. albopictus* mosquitoes. To test this hypothesis, we used the antagomiR approach to deplete miR-281 expression in vivo. Specific depletion of individual miRNAs by antagomiRs has been previously reported in mice and mosquitoes [20,26-29]. Female mosquitoes were injected with antagomiR-281 and control antagomiRs. Efficient knock-down of miR-281 was confirmed by qRT-PCR analysis in both mosquitoes and midguts. In female mosquitoes injected with miR-281 antagomiRs, the expression of miR-281 was significantly decreased (*F* =401.245, *P* <0.001) compared with control antagomiRs and normal mosquitoes (Figure 7A). A significant decrease of miR-281 expression (*F* =84.625, *P* <0.001) was also found in the anatagomiR-281-injected midguts (Figure 7B). The decrease of miR-281 treated with antagomiRs might have been due to degradation of the miRNA according to previous reports [26]. These results indicate the efficient depletion of miR-281 by means of antagomiR injection. To further confirm the effect of miR-281 knock-down on viral replication in vivo, the injected female mosquitoes were infected with DENV-2 at 48 hrs post-injection. Samples were collected and analyzed by RT-qPCR at days 1, 4 and 7 post-infection. As shown in Figure 8, no significant difference of DENV-2 gRNA was found in antagomiR-281-injected mosquitoes at day 1 post-infection (*F* =4.580, *P* >0.05). However, consistent with the results in C6/36 cells, DENV-2 gRNA was significantly decreased in antagomiR-281-injected mosquitoes compared with those of normal mosquitoes and control antagomiR-injected controls at day 4 (*F* =76.886, *P* <0.001) and day 7 (*F* =13.748, *P* <0.001) post-infection (Figure 8). The relative expression levels of miR-281 in which mosquitoes and midguts injected with miRNA antagomiRs and subsequently infected with DENV-2 were tested, and the results are shown in Figure 9. Levels of miR-281 were significantly lower in antagomiRs injected groups than in controls at 1–4 days after DENV-2 infection, which indicates that DENV-2 infection has little effect on the silencing of miR-281 in vivo. Thus, this in vivo analysis, in addition to the in vitro overexpression and knock-down experiments, indicates that the mosquito miR-281 miRNA enhances DENV-2 replication.

### MiR-281’s potential target is the 5′-UTR of DENV genome

**Figure 10:**
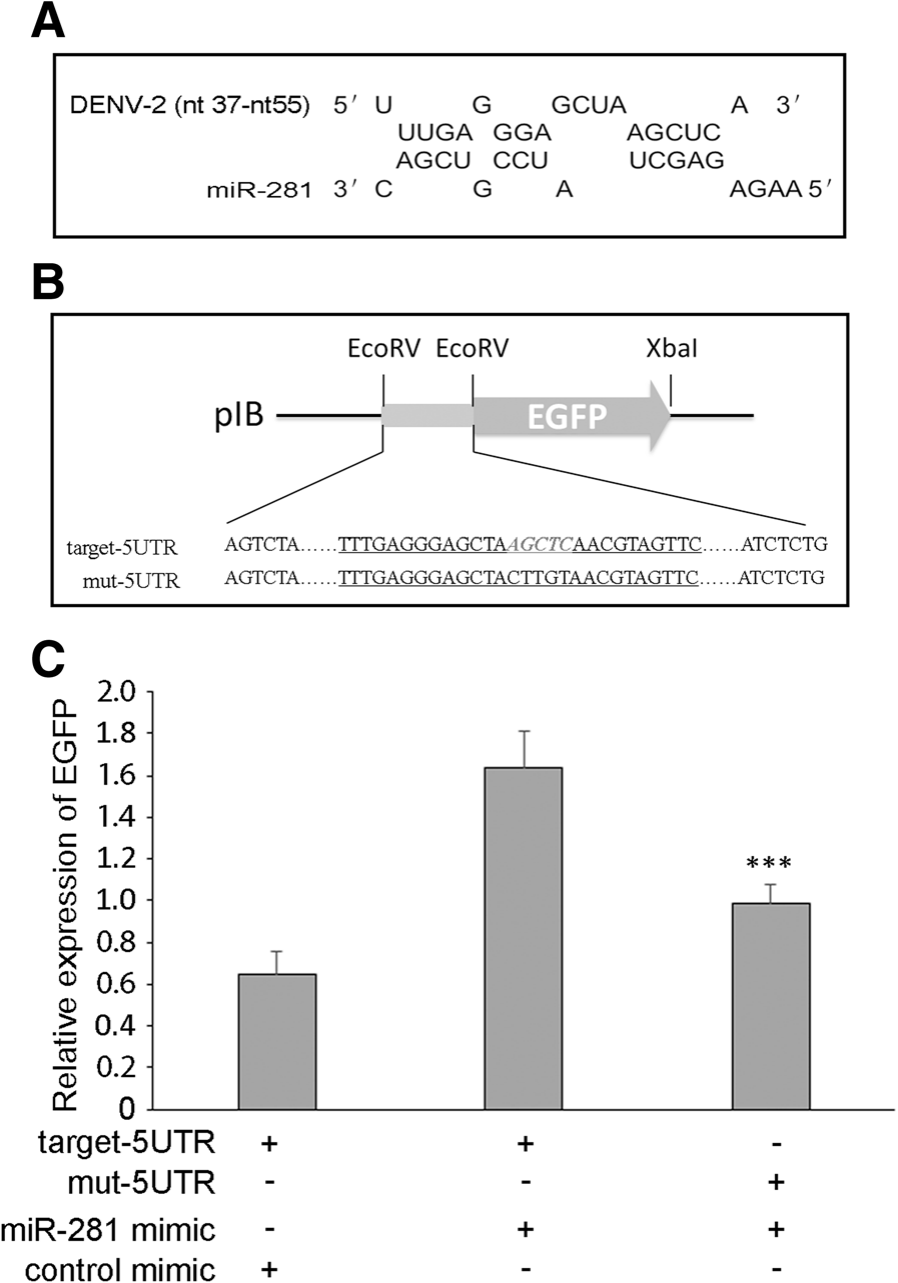
**MiR-281 target study. (A)** RNAhybrid prediction for miR-281 target sites and putative target sequences in the 5′-UTR of DENV-2 gRNA. **(B)** Cloning strategy of the 5′-UTR and mutant 5′-UTR upstream of the EGFP reporter gene ORF and downstream of pIB in the pIB/V5-His vector, denoted as target-5UTR and mut-5UTR. Target sequences are underlined, nucleotides complementary to the miR-281 seed region are shown in bold italic type. **(C)** Constructs were co-transfected with the miR-281 mimic or control mimic into C6/36 cells. Analysis by RT-qPCR shows that expression of EGFP was significantly higher in target-5UTR and miR-281 co-transfected cells than in mut-5UTR and miR-281 co-transfected as well as target-5UTR and control mimic-transfected cells. The RT-qPCR data were analyzed normalized to the blasticidin resistance gene. The RT-qPCR data were analyzed using the 2^-Δct^ method. Data shown represent three independent experiments with three triplicates each. *P* values are from ANOVA.

To explore whether miR-281 has a potential interaction with the DENV-2 genome, potential binding sites of miR-281 in the DENV-2 genome were predicted using RNAhybrid. Based on the mfe scores, one potential miR-281 target was found: nt37-nt55 (mfe = −21.4 kcal/mol), which is within the 5′-UTR SLA structure of the DENV-2 genome (Figure 10A). To investigate the interaction between the seed region of miR-281 and the predicted biding site in the 5′-UTR, we used an EGFP plasmid-based reporter system in the C6/36 cell line [22,30,31]. The DENV 5′-UTR and the mutant 5′-UTR sequence (see Methods) were cloned upstream of the EGFP gene in the pIB expression vector, resulting in the constructs pIB/5UTR-EGFP (target-5UTR) and pIB/mut-5UTR-EGFP (mut-5UTR), respectively (Figure 10B). The constructs were co-transfected with miR-281 or control mimics into C6/36 cells. Significantly higher EGFP transcript levels were detected in pIB/5UTR-EGFP and miR-281 mimic co-transfected cells compared with those cells co-transfected with pIB/5UTR-EGFP and control mimics (*F* =40.111, *P* <0.001). Moreover, pIB/mut-5UTR-EGFP and miR-281 co-transfected cells exhibited a significant decrease (*P* <0.001) in EGFP expression compared with pIB/5UTR-EGFP and miR-281 mimic co-transfected cells, while no significant difference (*P* >0.05) was found between pIB/5UTR-EGFP and control mimic co-transfected cells at 48 hrs post-transfection (Figure 10C).

## Discussion

In mosquitoes, midgut tissue plays important roles in DENV infection [32,33]. It is the first barrier for pathogen transmission. According to our previous study, miR-281 is a highly abundant miRNA in the mosquito vector *Ae. albopictus* [15]*.* Further sequencing analysis of miRNAs showed a high level of expression of miR-281 in both infectious blood-fed and normal blood-fed female midguts (unpublished, Yanxia Liu et al.). In this study, based on qPCR and Northern blot analyses, we found that miR-281 is highly specifically expressed in the midgut of the mosquito *Ae. albopictus* (Figure 1). *Ae. aegypti* miR-281*(called aae-miR-281-5p in miRBase), which is homologous to *Ae. albopictus* miR-281, was also found to dominate the miRNA content of the midgut [19]. Considering that an abundant liver-specific miR-122 modulates replication of the HCV viral RNA [12], the highly midgut-specific expression of mosquito miR-281 indicates its specific role in this tissue. Moreover, significant increases were observed in both DENV-2-infected mosquitoes and midguts, which indicated an involvement of miR-281 in the host-virus interaction. In addition, miR-281 was also found to respond to WNV infection in another important flavivirus vector mosquito, *C. quinquefasciatus* [13]. Thus, we presume that miR-281 mediates DENV-2 infection in the mosquito host.

Subsequent functional interventional studies of miR-281 showed that overexpression enhanced DENV-2 replication and down-regulation suppressed DENV-2 replication in C6/36 cells. Consistent with the in vitro result, depletion of miR-281 led to a decrease in DENV-2 gRNA in vivo. These findings suggest that miR-281 enhances DENV-2 replication by targeting the viral transcript or host mRNA.

Because the mosquito *Ae. albopictus* genome has yet to be sequenced, we searched for a potential target for miR-281 in the DENV-2 genome using computational software. The predicted target of miR-281 was located in the 5′-UTR SLA structure of DENV-2 (nt37–nt55), in a sequence that is conserved among different serotypes [34,35]. Although the predicted target did not match well with the putative seed sequence (nucleotides 2–7) of miR-281, it has consecutive matching nucleotides to miR-281 with a low mfe value. Several functional target sites have also been found to lack perfect seed pairing and 3′ compensatory binding in other studies [8,36]. Moreover, we utilized synthetic mimics and an EGFP-based reporter construct in the C6/36 cell line and found that a miR-281 mimic significantly enhanced the EGFP transcript level of pIB/5UTR-EGFP, while the expression of pIB/mut-5UTR-EGFP was not enhanced. These results indicate the interaction between miR-281 and the 5′-UTR sequence in the context of a plasmid-based reporter.

In our study, we found that miR-281 enhanced DENV-2 replication. In most cases, the interaction of a miRNA with its target results in the degradation of the target mRNA or the inhibition of translation [37]. However, there are reports of mRNA-miRNA interactions that can lead to the stability and up-regulation of mRNA. For example, miR-10a positively regulates global protein synthesis via the stimulation of ribosomal protein mRNA translation and ribosome biogenesis [38]. MiR-2940, which is induced after *Wolbachia* infection, up-regulates the metalloprotease gene of *Ae. aegypti* [39]. In the mosquito midgut, an important tissue that plays a role in DENV transmission, it is critical for DENV-2 to successfully establish infection in this tissue first in vivo, and it is then transmitted to other tissues. The high expression of miR-281 in the midgut allows DENV-2 to exploit this host cell factor to the benefit of its replication. There is an emerging belief that RNA viruses have retained target sites for cellular miRNAs to benefit the viral life cycle in some manner [40]. Indeed, the replication of two RNA virus, coxsackie virus B3 and HCV, has been reported to be promoted by the host cellular miRNAs miR-10a* and miR-122, respectively [11,12,41]. For miR-281, in the current study, we propose that miR-281 may enhance DENV-2 replication by targeting the 5′-UTR of DENV-2 gRNA. To confirm the hypothesis, further study is needed to confirm the direct interaction between miR-281 and DENV-2 genome.

## Conclusion

In conclusion, miR-281 has been found to be specifically expressed at a high level in the midgut of female adult mosquitoes and is up-regulated after DENV-2 infection. Functional interventional studies confirm that miR-281 enhances DENV-2 replication. A target study indicates that there is an interaction between miR-281 and the DENV-2 5′-UTR sequence in the context of the EGFP reporter. To our knowledge, this is the first report of a midgut-specific miRNA that is involved in DENV replication. Further study of the reverse genetic experiments would be performed to confirm the direct interaction between miR-281 and DENV-2 genome.

## Additional file

Additional file 1: Table S1. qPCR primers used in this study.
