# Supplementary material for: miR-281, an abundant midgut-specific miRNA of the vector mosquito Aedes albopictus enhances dengue virus replication
Source: Parasit Vectors. 2014 Oct 22;7:488. doi: 10.1186/s13071-014-0488-4 (PMC4212107; doi:10.1186/s13071-014-0488-4)
Supplement: Additional file 1: Table S1. — qPCR primers used in this study. [file 13071_2014_488_MOESM1_ESM.doc]

Additional file 1: Table S1 qPCR primers used in this study

| Primer name | Primer sequence(5'-3') |
| --- | --- |
| miR-281 | 5'-AAGAGAGCTATCCGTCGACAGT-3' |
| 5S rRNA | 5'-TCGCGTGTCGTTGGCCAA-3' |
| EGFPF | 5'-GGACGACGGCAACTACAAGA-3' |
| EGFPR | 5'-TCTGCTTGTCGGCCATGATA-3' |
| blasticidin resistance gene F | 5'-ATTTTACTGGGGGACCTTGC-3' |
| blasticidin resistance gene R | 5'-AGCAATTCACGAATCCCAAC-3' |
